# Potential Link Between a Disruptive *CAPN6* Variant and Neurodevelopmental Disorders

**DOI:** 10.3390/ijms27031140

**Published:** 2026-01-23

**Authors:** Francesco Calì, Simone Treccarichi, Mirella Vinci, Emanuela Avola, Antonino Musumeci, Alda Ragalmuto, Carola Costanza, Donatella Greco, Desiree Brancato, Concetta Federico, Santina Città, Francesco Domenico Di Blasi, Salvatore Saccone, Paolo Scudieri, Federico Zara, Maurizio Elia

**Affiliations:** 1 Oasi Research Institute-IRCCS, 94018 Troina, Italy; cali@oasi.en.it (F.C.); streccarichi@oasi.en.it (S.T.); eavola@oasi.en.it (E.A.); amusumeci@oasi.en.it (A.M.); aragalmuto@oasi.en.it (A.R.); ccostanza@oasi.en.it (C.C.); dgreco@oasi.en.it (D.G.); scitta@oasi.en.it (S.C.); fdiblasi@oasi.en.it (F.D.D.B.); melia@oasi.en.it (M.E.); 2 Department of Medicine and Surgery, Kore University of Enna, 94100 Enna, Italy; 3 Department of Biological, Geological and Environmental Sciences, University of Catania, 95124 Catania, Italy; desiree.brancato@phd.unict.it (D.B.); concetta.federico@unict.it (C.F.); 4 Medical Genetics Unit, IRCCS Istituto Giannina Gaslini, 16147 Genoa, Italy; paolo.scudieri@unige.it (P.S.); federico.zara@unige.it (F.Z.); 5 Department of Neurosciences, Rehabilitation, Ophthalmology, Genetics, Maternal and Child Health (DiNOGMI), University of Genoa, 16147 Genoa, Italy

**Keywords:** calpain 6, placental developmental disorders, next generation sequencing, vascular endothelial growth factor

## Abstract

The placenta is often described as the “window to the brain” due to its crucial role in fetal neurological development. In this study, we investigated a family where the older male offspring exhibited severe neurodevelopmental and mild motor coordination disorders. His brother displayed emotional and behavioral dysregulation along with mild motor coordination disorders. The father was asymptomatic, while the mother and daughter showed mild learning disabilities. Whole exome sequencing (WES) identified a disruptive X-linked pathogenic variant, c.1088_1089del p.Asp363GlyfsTer2, within the calpain-6 (*CAPN6*) gene. We have submitted this variant to the ClinVar database (RCV005234146.2). The variant was found in hemizygous condition in the affected male offspring and in heterozygous condition in both the mother and daughter. As predicted, the variant undergoes nonsense-mediated mRNA decay (NMD), preventing the translation of the *CAPN6* gene into a functional protein. *CAPN6* is a critical gene predominantly expressed in placental and trophoblast tissues. Although its function is not well characterized, CAPN6 is also expressed in several regions of the developing brain. Recent studies have shown that genetic variants in *CAPN6* significantly influence vascular endothelial growth factor (VEGF) activity, thereby affecting angiogenesis and the blood supply essential for fetal growth and development. Although *CAPN6* lacks an MIM phenotype code, we hypothesize that it might be enumerated as a novel candidate gene contributing to neurodevelopmental disorders. Functional studies are imperative to elucidate the role of *CAPN6* in placental function and its potential implications for neurodevelopmental processes. This work aims to inspire further research into the role of *CAPN6* in placental biology and its relevance to neurodevelopmental disorders.

## 1. Introduction

The placenta plays a pivotal role in fetal development by facilitating nutrient exchange, oxygenation, and waste removal, which are essential for fetal growth. Disruptions or genetic abnormalities affecting placental function may contribute to neurodevelopmental disorders in the fetus. Placental issues or mutations in placental genes may impact fetal brain development, potentially increasing the risk of conditions like autism spectrum disorder (ASD) or intellectual disability (ID), highlighting the significance of a healthy placenta for optimal neurodevelopment [1,2,3]. Within this context, the placenta has been described as a “window to the brain” owing to accumulating evidence supporting its critical role in prenatal neurodevelopmental programming. Indeed, gene–environment interactions during pregnancy may contribute to the development of mental disorders later in life [3,4]. In the placenta, fetal DNA acts as a foundational genetic plan essential for the organ’s formation and function, mirroring the genetic identity of the developing fetus. Alongside DNA, fetal RNA expression within the placenta coordinates a range of developmental processes, such as tissue differentiation and hormone production, which are essential for sustaining pregnancy [5,6].

The calpain–calpastatin system in the human placenta plays a pivotal role in regulating proteolytic activity essential for placental development and function. Calpains, calcium-dependent cysteine proteases, and their endogenous inhibitor calpastatin are expressed in both fetal and maternal placental tissues. This system ensures proper cellular processes, such as trophoblast invasion and angiogenesis, highlighting the complex interplay between fetal and maternal gene expression required to maintain a healthy pregnancy and ensure embryonic survival [7,8].

Among the genes primarily expressed in the diverse placenta tissues involved in the calpain–calpastatin system, calpain-6 (*CAPN6*) emerges as a key component, involved in multiple biological functions. As a member of the calpain protein family, calpain-6 (*CAPN6*) is highly expressed mainly in the placenta and embryos. In contrast to most other calpain family members, *CAPN6* exhibits non-proteolytic activity [9]. It plays a number of important roles in cellular processes. Specifically, acting as a molecular chaperone, it contributes to microtubule stabilization. Furthermore, it actively operates in the maintenance of cell stability, the control of cell movement and the inhibition of apoptosis. The engagement of CAPN6 in microtubule dynamics and actin reorganization was studied using *Capn6*-deficient mice. Within this context, it was demonstrated that CAPN6 suppresses skeletal muscle differentiation during development and regeneration [10,11]. It was found to be predominantly expressed during embryogenesis in developing skeletal and cardiac muscle [7,12]. Furthermore, *CAPN6* plays a crucial role in osteoclast function by localizing to the sealing zone, where it promotes actin ring formation and bone degradation when overexpressed. Conversely, *CAPN6* knockdown disrupts cytoskeletal organization and impairs the cell’s ability to resorb bone, partly through its interaction with tubulin, which affects microtubule stability and acetylation [13]. As outlined, *CAPN6* facilitates cytoskeletal organization and microtubule stability in osteoclasts, suggesting its inhibition might contribute to the resorption-arresting effects of glucocorticoids [13].

As was outlined by various studies, high expression of *CAPN6* contributes to the tumorigenesis of uterine sarcomas and carcinosarcoma. In particular, CAPN6 actively orchestrates tumor development by promoting cell proliferation and angiogenesis, and by inhibiting apoptosis [14,15].

Calpain-6 has been identified as a positive regulator of ciliogenesis [16]. Its deficiency leads to a decrease in ciliated cell percentage and disrupts Sonic Hedgehog signaling (SHH). This effect may be associated with reduced α-tubulin acetylation at lysine 40, representing the first evidence of calpain-6 involvement in primary cilia formation.

*CAPN6*, due to its abnormal cellular expression, has been linked to several diseases, including white matter damage and muscular dystrophy, making it a potential novel therapeutic marker [9].

In the current study, whole exome sequencing (WES) revealed a deletion within the *CAPN6* gene. This mutation was found in three siblings who experienced global developmental delay (GDD) in early childhood. While the father was unaffected, the mother also showed a specific learning disorder (SLD), and borderline intellectual functioning (BIF). Given the predominant expression of *CAPN6* in placental tissues and its additional expression in the developing brain, this study explores a potential link between CAPN6 dysfunction and neurodevelopmental outcomes. Although causality cannot yet be established, our findings support the hypothesis that *CAPN6* may represent a novel candidate gene contributing to neurodevelopmental disorders, possibly through placental-mediated mechanisms.

## 2. Results

### 2.1. Clinical Reports

In the following sections, we describe the clinical profiles of the three offspring and their mother. Patient 1, the eldest sibling (male); Patient 2, the middle child (female); and Patient 3, the youngest sibling (male) all presented with GDD during early childhood. From a neurodevelopmental perspective, their diagnostic trajectories have been longitudinally monitored, revealing different clinical trajectories over time. In particular, the progression of the initial developmental delay has shown variable prognostic implications, with some patients experiencing a more severe deterioration over time. Detailed clinical, neuropsychological, and neurodevelopmental assessments of these patients, as well as their family history and the maternal clinical profile, are provided in the subsequent sections.

### 2.2. Family History

All examined individuals presented with a probable family history of schizophrenia and intellectual disability (ID) on the maternal side. However, a comprehensive evaluation was not feasible, as the relatives did not consent to further investigation by our institution. Notably, the maternal great-grandmother was reported to have schizophrenia, and the maternal cousin was described as affected by both schizophrenia and intellectual disability. Although these diagnoses have not been formally confirmed, the reported clinical features—particularly the prolonged presence of hallucinatory phenomena in the absence of pharmacological treatment—are suggestive of schizophrenia.

#### 2.2.1. Patient 1

Patient 1 is the first-born male, born at 37 weeks of gestation via eutocic delivery, with a birth weight of 3050 g, a length of 49.5 cm, and a head circumference of 34 cm. From early infancy, he exhibited GDD, attaining independent walking at 18 months, beginning to babble at 12 months, and uttering his first words at 2 years of age.

At 2 years, the patient experienced complex febrile seizures (CFS), which were effectively managed with valproic acid (VPA) at 30 mg/kg/day, achieving seizure control. Concurrently, his phenotype was notable for generalized muscular hypotonia, ligamentous laxity, a small café-au-lait spot on the trunk, clinodactyly of the fourth and fifth toes, winged scapulae, dorsal hyperkyphosis, and tibial varus deformity. Although no focal neurological deficits were detected, he demonstrated impairments in both gross and fine motor coordination and exhibited oppositional-defiant behaviors. Extensive molecular investigations—including array-CGH, karyotyping, SCN1A sequencing, and FRAXA region analysis—yielded normal results.

**Figure 1:**
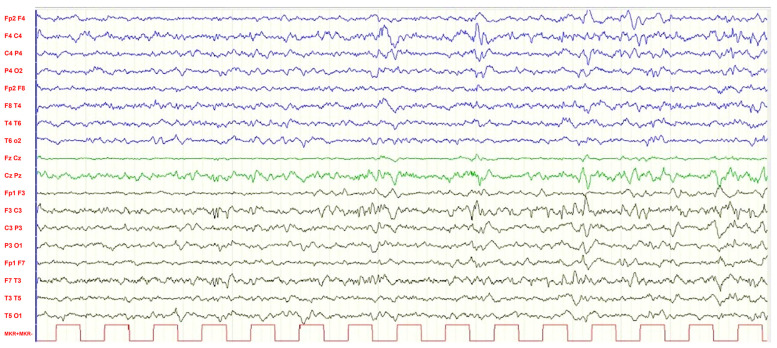
Electroencephalography (EEG) conducted in Patient 1. The analysis demonstrated a predominant parieto-occipital alpha rhythm (11 Hz) and moderate amplitude spikes in the central regions during sleep.

By the age of 7 years, his neurodevelopmental trajectory had evolved, and he was diagnosed with moderate intellectual disability with WISC IV [17]: IQ = 41 and adaptive behavior ABAS II [18] = 40. At this stage, he presented with marked microcephaly (approximately four standard deviations below the mean), a height of 126 cm (75th–90th percentile), and a weight of 21 kg (25th percentile). His behavioral profile deteriorated, manifesting increased impulsivity, inappropriate language, and hetero-aggressive behaviors, which were managed with risperidone solution at 1 mL/day. Brain magnetic resonance imaging (MRI) was unremarkable, while electroencephalography (EEG) demonstrated a predominant parieto-occipital alpha rhythm (11 Hz) and moderate amplitude spikes in the central regions during sleep (Figure 1).

At 10 years of age, further neuropsychiatric evolution was observed. The patient developed self-injurious behaviors, bizarre conduct, a tendency to steal, disorganized speech, visual perceptual disturbances (including hallucinations of insects) and specific phobias. In response, his therapeutic regimen was modified by introducing Levomepromazine at 50 mg/day and increasing the VPA dosage to 700 mg/day, which resulted in significant improvement of his symptoms.

Currently, at 18 years, the patient exhibits a marfanoid habitus with persistent microcephaly and distinctive craniofacial features. His face is oval and elongated, with a narrow biparietal diameter, a slightly sloping forehead, narrow palpebral fissures, a prominent nasal tip, a long and poorly defined philtrum, and a thin upper lip. His clinical phenotype also includes a macroorchidism. Neurological examination reveals a head circumference of 51.4 cm, muscular hypotonia with preserved muscle trophism, symmetric deep tendon reflexes, absence of a pathological Babinski sign bilaterally, and a broad-based gait with mild external rotation of the feet. Psychiatrically, he now presents with a complex profile characterized by moderate intellectual disability with adaptive behavior ABAS II [18] = 40, accompanied by a schizophrenia spectrum disorder (SSD). Persistent behavioral disturbances such as impulsivity, irritability, poor adherence to social norms, and intolerance to frustration are evident, along with altered thought content manifested by fixed ideas, repetitive speech, and soliloquies (described as speaking about imaginary entities, engaging in aimless movements, gesticulating, occasionally shouting, and using profane language). Episodes of familial aggression are also noted. Notably, EEG findings at this stage do not reveal any epileptic discharges or specific abnormalities, prompting the discontinuation of VPA therapy. His current pharmacological regimen consists of risperidone solution at 1 mL/day and Levomepromazine at 50 mg/day.

#### 2.2.2. Patient 2

The patient is a female, the second-born child, delivered at 38 weeks of gestation via spontaneous delivery following an uneventful pregnancy. At birth, her weight was 2800 g and her length 48 cm. From early infancy, she exhibited delayed developmental milestones, attaining independent walking at 16 months, initiating babbling at 12 months, and achieving sphincter control by 3 years of age. She was initially diagnosed with GDD.

At 5 years of age, anxiety-related features began to emerge. Although she had acquired language, she was diagnosed with a Speech Sound Disorder (SSD). Electroencephalography (EEG) yielded normal findings, while electrocardiography (ECG) revealed a prolonged QTc interval of 0.44 s. Brain magnetic resonance imaging (MRI) was unremarkable, and genetic investigations, including chromosomal microarray (CGH-array), showed no abnormalities. Menarche occurred at 11 years of age, with subsequent regular menstrual cycles.

Currently, at 14 years of age, the patient presents with a normal IQ (IQ WISC IV [17] = 93); however, she meets the criteria for an SLD affecting reading, writing, and arithmetic skills. Additionally, she suffers from recurrent headaches responsive to non-steroidal anti-inflammatory drugs (NSAIDs). An orthopedic evaluation, including foot radiography, revealed hypoplasia in the length of the fourth metatarsal. Neurological examination demonstrated normal muscle tone and trophism, a normal gait, and no deficits in balance, while language and socialization skills are now appropriate for her age.

Her psychiatric profile is notable for anxious traits and a tendency to somatize internal emotional states, manifesting as abdominal pain and a persistently low mood, although these symptoms do not fulfill the criteria for a clinical anxiety disorder or depression and are thus classified as internalizing disorders. No episodes of suspected epileptic activity have been reported.

#### 2.2.3. Patient 3

Patient 3 is the third-born male child, delivered at 37 weeks and 5 days via induced labor due to an increased risk of placental abruption. His birth weight was 3150 g, and his length was 47 cm. From early infancy, he exhibited delayed developmental milestones, achieving independent walking at 17 months with minimal language development. Plagiocephaly was noted at birth.

At 2 years of age, an electroencephalogram (EEG) revealed poor organization and slow background activity. He had a diagnosis of global developmental delay (GDD) and initial behavioral problems. By the age of 4, Patient 3 began to exhibit significant behavioral issues, including marked irritability, poor adherence to rules, and aggressive behaviors. He was diagnosed with Language Disorders (LD) and developmental coordination disorder (DCD). Intellectual functioning was evaluated as normal (IQ Leiter 3 [19] = 89). Despite these concerns, brain magnetic resonance imaging (MRI) was unremarkable, and genetic investigations—including chromosomal microarray (CGH-array) and karyotype analysis—yielded normal results. Testing for SCN1A and FRAXA mutations also excluded chromosomal anomalies and nucleotide expansions, while repeated neurological examinations have consistently revealed no abnormalities. To further explore the potential underlying cause of the patient’s clinical phenotype, a WES Trio analysis has been scheduled.

Currently, at 7 years of age, the patient has been diagnosed with Borderline Intellectual Functioning (BIF) with an IQ Leiter 3 [19] = 79, Oppositional Defiant Disorder (ODD), LD, and DCD. He also exhibits marked hyperkinesia and hyperactivity. Notably, his oppositional behaviors are similar to those observed in his older brother, although his intellectual functioning remains only slightly below the normative average for his age and standardized peers. While the future evolution of his phenotypic and behavioral profile remains uncertain, the severity of his behavioral disturbances necessitated the initiation of treatment with risperidone solution at 1 mL/day, which has shown promising efficacy.

#### 2.2.4. Patient 4—Mother

Patient 4 is a 37-year-old female with borderline intellectual functioning (WAIS-IV [20] IQ = 71) and an SLD affecting reading, writing, and arithmetic. Her adaptive functioning is within normal limits, and she showed normal acquisition of psychomotor developmental milestones. Initial learning difficulties were noted at age seven and formally assessed at age 11; she left school during the first year of secondary education. At 37, she also exhibits significant mood disturbance and anxiety, with elevated scores on both the Beck Depression Inventory and the Beck Anxiety Inventory [21].

A comprehensive comparison of the three examined siblings with their mother is presented in Table 1.

### 2.3. Next-Generation Sequencing (NGS)

**Figure 2:**
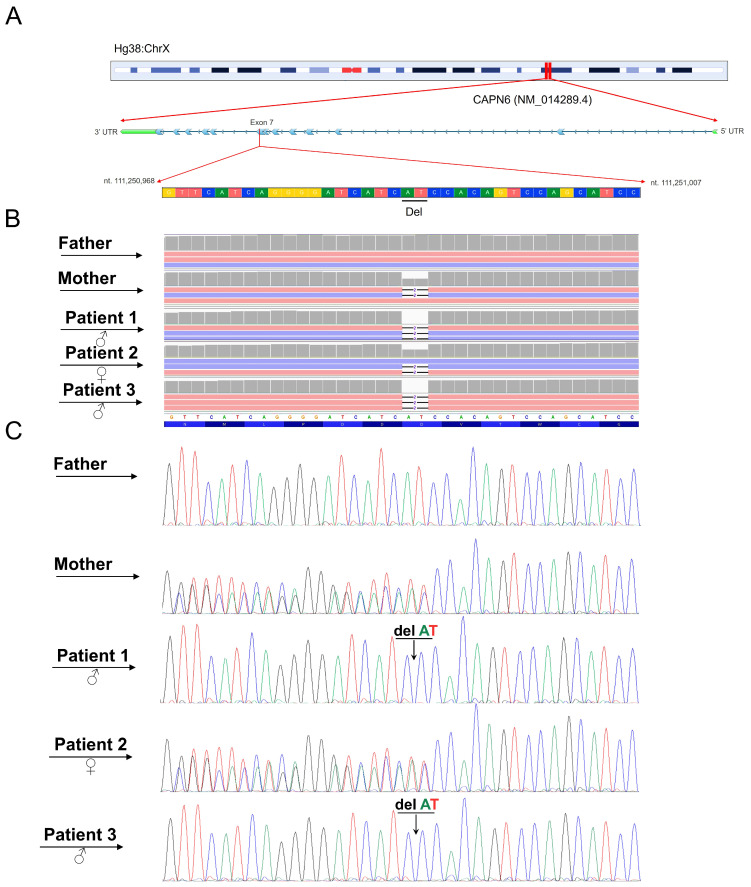
NGS analysis identified the variant c.1088_1089del within the *CAPN6* gene (NM_014289.4) in the family examined. (**A**) Both the chromosomal and exon localization of the CAPN6 gene and variant are depicted. (**B**) The Integrative Genomics Viewer (IGV) image displays this variant, with arrows indicating the father, mother, and their three sons. (**C**) Confirmation of the variant through conventional Sanger sequencing is presented for the father, mother, and their three sons, as indicated by the black arrows. Specifically, the variant has been found in hemizygous condition in the affected male offspring while in heterozygous condition for both the mother and daughter.

WES analysis identified a novel genetic variant (c.1088_1089del) within the *CAPN6* (NM_014289.4) gene (Figure 2).

**Figure 3:**
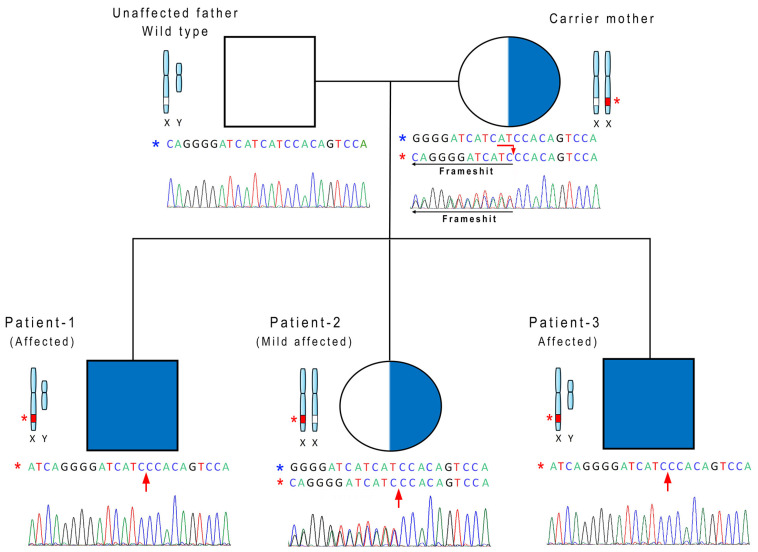
Pedigree analysis of the examined family with an X-linked genetic disorder associated with the mutation c.1088_1089del (p.Asp363GlyfsTer2) in the *CAPN6* (NM_014289.4) gene. Both the mother and the daughter exhibited mild intellectual disability. Furthermore, both the male sons exhibited severe neurodevelopmental and mild motor coordination disorders. Red arrows indicate the specific position of the variant. Blue and red asterisks indicate wild type and mutated sequence, respectively.

The variant is located on the X chromosome and segregates within the family as shown in Figure 3.

The identified variant was classified as pathogenic according to ACMG criteria (PP1, PVS1 and PM2) and submitted to the ClinVar database (RCV005234146.2). No additional variants were identified in known disease-associated genes. Furthermore, the Mutation Taster algorithm classified the variant as disease-causing. Conventional Sanger sequencing confirmed the presence of this mutation in the affected family members.

NMDEscPredictor indicated that the identified mutation (p.Asp363GlyfsTer2) is predicted to trigger nonsense-mediated mRNA decay (NMD).

According to the gnomAD database, the variant exhibits a very low allele frequency (f = 0.000001651) in the world population (1,211,311 alleles). Specifically, the variant has been found only in 2 alleles belonging to the European non-Finnish ethnicity group (f = 0.000002234). Notably, in this mentioned ethnicity group, it was found in hemizygous condition in one male (XY), and in heterozygous condition in one female (XX). Additionally, the specific mutation site displays a moderate conservation rate, according to the conservation score parameter PhyloP (3.895). Although the variant has been reported in gnomAD, no associated phenotypic information is available, limiting conclusions regarding penetrance and expressivity.

**Figure 4:**
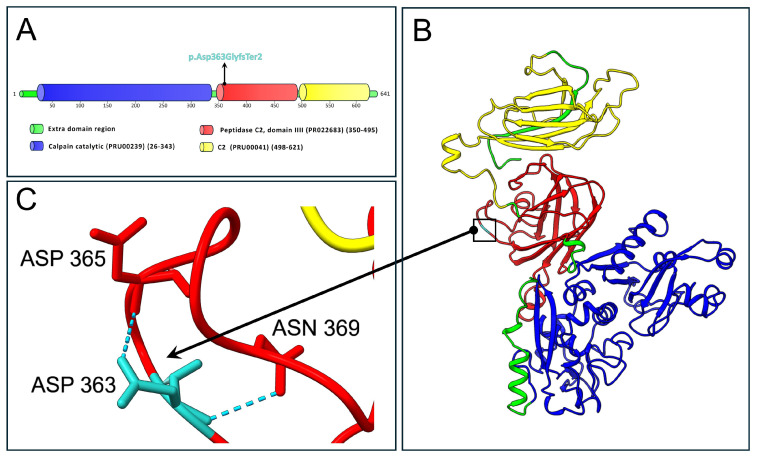
Domain organization and structural prediction of wild-type CAPN6. (**A**) Schematic representation of the functional domains of CAPN6, annotated according to the ProRule and InterPRO databases. A black arrow marks the mutation site at position 363 in the amino acid sequence. (**B**) Structural model generated using AlphaFold3, with domains colored according to panel. (**A**) The mutation site (Asp363) is highlighted in turquoise and indicated by a black square. (**C**) Close-up view of the wild-type residue (turquoise) and its hydrogen-bonding interactions with Asp365 and Asn369. Structural visualization and modeling were performed using UCSF ChimeraX.

Figure 4 depicts the protein structure of CAPN6 with a specific close-up view on residue 363.

## 3. Discussion

In the current manuscript, we describe a family in which all three siblings exhibited GDD in early childhood. Both male siblings carry the same hemizygous disruptive mutation in the *CAPN6* gene, whereas the female sibling displays a milder phenotype characterized solely by language disorder (LD). She also has a learning disorder like her mother. They are heterozygous for the variant. The unaffected father does not carry the mutation. The identified variant, p.Asp363GlyfsTer2, results in the substitution of aspartate with glycine at position 363, followed by a premature stop codon. This variant has been classified as pathogenic in accordance with ACMG criteria, as detailed in Table 1. The patients’ phenotype displays vertical transmission consistent with an X-linked inheritance pattern with variable expressivity, in which the affected sons inherited the disorder from a mildly affected heterozygous mother. The marked phenotypic variability observed among carriers of the *CAPN6* variant, including differences in severity between the affected male siblings and milder manifestations in heterozygous females, suggests variable expressivity rather than a uniform clinical outcome. Such variability may be influenced by multiple factors, including genetic modifiers elsewhere in the genome, additional neurodevelopmental risk variants segregating within the maternal lineage, or differences in X chromosome inactivation patterns in heterozygous females. Moreover, environmental and stochastic factors—particularly those related to the prenatal environment and placental function—may further modulate phenotypic expression. Together, these considerations underscore that the identified *CAPN6* variant should be viewed as a potential contributor within a multifactorial context rather than as a sole causative determinant of the observed neurodevelopmental phenotypes. Notably, the *CAPN6* gene was not flagged by the DOMINO inheritance predictor, suggesting an unconventional or previously unrecognized role in disease transmission. This discrepancy highlights the still poorly understood role of CAPN6 in disease origin and inheritance. To date, the *CAPN6* gene has never been associated with a specific phenotype through an MIM phenotype code [22]. Importantly, this gene has not been previously linked to any neurodevelopmental disorder but rather to uterine cancer [15].

An interesting aspect is that the mother has a family history of schizophrenia and intellectual disability. However, we were unable to conduct genetic analysis on her relatives. Despite this, we cannot rule out the possibility that other genetic factors, which are not detectable by WES—such as mutations in non-coding regions or polygenic risk factors involving inter-allelic complementation—may contribute to the patient’s phenotype. Chromosomal anomalies and nucleotide expansions were excluded through array-CGH, karyotype analysis, and testing for SCN1A and FRAXA mutations, all of which yielded normal results. To further investigate the potential underlying cause of the patient’s clinical features, a WES Trio analysis has been scheduled. Within this context, we consider it unlikely that environmental or epigenetic factors alone account for the development of the patients’ phenotypes, as it would be statistically improbable for all three children to exhibit similar psycho-behavioral features solely due to such influences. Additionally, the mother’s mild symptoms as well as the heterozygous condition support the exclusion of this hypothesis. Therefore, in this manuscript, we aim to explore the potential correlation between the *CAPN6* gene, and the symptoms observed in the examined family.

The inheritance pattern suggests that *CAPN6* is the implicated gene, as it is located on the X chromosome. According to the principles of non-Mendelian inheritance related to X-linked transmission, this hypothesis seems consistent. The mother and her daughter, both carriers of the genetic variant, exhibit mild symptoms due to the presence of one additional X chromosome compared to the affected males [23]. The older brother has a severe intellectual disability, while the younger brother has been diagnosed with moderate-to-severe developmental delay. However, as the younger brother is only seven years old, we do not rule out the possibility of a progressive worsening of his condition.

Previous studies identified a plausible correlation between mutations within calpain genes with neurodevelopmental disorders [24,25]. Particularly, calpain-1 was found to be intricately involved in neurogenesis [26,27,28]. As previously documented, a de novo mutation in the *CAPN6* gene was identified in a female patient presenting with severe symptoms that were Rett syndrome-like, including motor coordination disorders and profound intellectual delay. Although the authors attributed the syndrome to a de novo deletion in the *SHANK3* gene, the *CAPN6* variation was classified as “probably damaging” based solely on in silico computational predictions and was not considered causative of the clinical presentation [29]. Given this classification, we hypothesize that mutations in the *CAPN6* gene may also contribute to the neurodevelopmental disorders observed in this patient [29].

According to expression data from the BrainSpan database, *CAPN6* shows consistently low transcript levels across most brain structures throughout development. The heatmap depicted in Figure S1 illustrates the developmental expression profile of CAPN6 across different brain structures. However, its expression peaks transiently during neurogenesis, suggesting a potential role in neuronal generation. This pattern aligns with prior findings linking *CAPN6* to early developmental processes, including its *SHH*-dependent regulation in the ventral forebrain and ciliogenic function.

Calpain-6 (*CAPN6*) has been identified as a positive regulator of ciliogenesis, with its deficiency impairing primary cilium formation, reducing the proportion of ciliated cells, and disrupting Sonic Hedgehog (SHH) signaling [16]. This defect is linked to decreased acetylation of α-tubulin, highlighting *CAPN6* as a previously unrecognized modulator of ciliary dynamics. Its developmental expression pattern further supports a role in SHH signaling: in addition to its known expression in the first pharyngeal arch [11], recent studies have shown *CAPN6* expression in the SHH-responsive floorplate of the ventral forebrain as early as embryonic day [30]. Notably, *CAPN6* expression is absent in the ventral telencephalon and diencephalon of *Shh*–/– embryos, but retained in the pharyngeal arch—supporting its regulation by SHH. Based on these findings, we hypothesize that the observed mutation may impair neurogenesis, potentially by disrupting *CAPN6*-related cellular functions. However, this remains speculative, as the functional impact of the mutation cannot currently be experimentally verified.

Analysis of Human Protein Atlas (HPA) data reveals high expression of CAPN6 in human placenta. Figure S2 demonstrates consistent expression patterns across multiple female placental samples from different donors, with quantitative levels reported in normalized Transcripts per Million (nTPM). These findings are further supported by the expression profiling by microarray data from the GSE9984 dataset (Figure S3), which examined gene expression profiles in first-trimester, second-trimester, and term placental tissues using Affymetrix chips. Together, these complementary datasets establish *CAPN6* as prominently expressed throughout placental development. In this context, placenta has been defined as a “window in the brain” because of its critical involvement in prenatal neurodevelopmental programming. This is underscored by the notion that gene–environment interactions during pregnancy may contribute to the onset of mental disorders in adulthood [3]. *CAPN6* mRNA is highly expressed in the placental chorionic plate and is particularly prominent during embryogenesis in tissues such as the somite, mandibular tissue, developing skeletal muscle, myocardium, epithelial cells of the fourth ventricle, bronchial epithelium, lung buds, and kidneys [7,31,32]. As previously documented, defects in CAPN6 protein might lead to fetal growth restriction (FGR), resulting in reduced fetal size and weight in utero [32]. Infants born with FGR are at a higher risk of fetal asphyxia and neurodevelopmental disorders with a potential impact on brain structure [33,34]. Staining of calpain-6 and calpastatin was observed in the cytoplasm of trophoblast cells in both FGR and control placentas. In fact, it was observed that the expression of calpain-6 was significantly lower (*p* = 0.01) in FGR placentas [32]. CAPN6 was discovered to be upregulated in human induced pluripotent stem cells (hiPSCs) within a cellular model mimicking placental conditions (Tsuchida [35]). Conversely, CAPN6 expression was found to be significantly downregulated following the differentiation of trophoblast giant cells (TGCs) [36]. While no indices of fetal growth are available, it is noteworthy that the pregnancies of the male patients concluded at 37 weeks—falling within the early term period (typically defined as deliveries occurring between 37 0/7 and 38 6/7 weeks of gestation)—whereas the pregnancy of the female patient reached 38 weeks, which in this context is considered term. According to gene ontology annotations from the QuickGO database, CAPN6 plays essential roles in various biological activities. It does not exhibit calcium-dependent cysteine-type endopeptidase activity (GO:0004198), nor is it involved in proteolysis (GO:0006508), protein binding (GO:0005515), or microtubule binding (GO:0008017). However, CAPN6 is crucial for microtubule bundle formation (GO:0001578) and the regulation of cytoskeleton organization (GO:0051493). It is localized to the cytoplasm (GO:0005737), spindle (GO:0005819), cytoskeleton (GO:0005856), microtubule (GO:0005874), spindle microtubule (GO:0005876), and the perinuclear region of the cytoplasm (GO:0048471). The catalytic domain of the CAPN6 protein, annotated with Prosite and ProRule (PRU00239), demonstrates three primary molecular functions: cysteine-type peptidase activity (GO:0008234), hydrolase activity (GO:0016787), and peptidase activity (GO:0008233).

The NMDEscPredictor tool indicated that the identified variant is likely to trigger nonsense-mediated decay (NMD) of the *CAPN6* transcript, potentially resulting in transcript degradation and loss of protein production. This may suggest that a loss of CAPN6 function, with a possible contribution to impaired neurogenesis during placental development. However, we underscore that this is an in silico prediction and might not reflect the actual biological outcome. According to the Mutalyzer tool, if the transcript escapes NMD and is translated, the resulting protein would consist of 363 amino acids—significantly shorter than the 641 amino acids in the wild-type. The variant p.Asp363GlyfsTer2 replaces the aspartic acid at position 363 with glycine, introducing a premature stop codon. As a result, the truncated protein would lack both the peptidase C2 domain (residues 350–495) and the C2 domain (residues 498–621). AlphaFold3 structural predictions further support these findings. In the wild-type model, Asp363 forms two stabilizing hydrogen bonds with Asp365 and Asn369. These interactions are lost in the mutant Gly363, which does not participate in any hydrogen bonding. Figure S4 shows the prediction realized for the truncated CAPN6, highlighting on the residue Gly363. A comparison of the “best models” generated by AlphaFold3 for both wild-type and mutant CAPN6 revealed a substantial difference in the total number of hydrogen bonds: 598 in the wild-type versus 317 in the p.Asp363GlyfsTer2 mutant. Although these differences may vary depending on the specific prediction model and associated predicted local distance difference test (pLDDT) scores, we propose that, if expressed, the mutant CAPN6 protein would exhibit significant structural alterations relative to the wild-type.

As documented, CAPN6 interacts in the placental tissue with the vascular endothelial growth factor (VEGFA) in placental tissue. Yeast two-hybrid screening confirmed this interaction. Specifically, the CAPN6 domain from Gly321 to Asp500 binds to the C-terminus of VEGFA, which is essential for VEGF secretion and angiogenesis [37]. As documented, CAPN6 lacking this domain failed to promote VEGF secretion, highlighting its importance in the angiogenic process. VEGF is critical for human placental development, influencing angiogenesis, trophoblast invasion, and spiral artery remodeling, all necessary for proper fetal growth and development [38].

As previously emphasized, the mutation described in this study may lead either to degradation of the *CAPN6* mRNA transcript via nonsense-mediated decay or to the production of a truncated protein lacking the region from residue 364 to the C-terminus. In both scenarios, these findings raise the possibility that the interaction between CAPN6 and VEGFA may be impaired. To explore this, we compared AlphaFold3-predicted interaction models for both wild-type and mutant CAPN6 in complex with VEGFA. Supplementary Figure S5 presents the top-ranked model of the wild-type CAPN6–VEGFA interaction, with the corresponding hydrogen bond interactions detailed in Table S1. The analysis identified key hydrogen bonds that are potentially lost or altered in the mutant model, suggesting a disrupted binding interface. The predicted structural outcome of the CAPN6–VEGFA interaction in the mutant context is shown in Supplementary Figure S6, while Table S2 lists the predicted hydrogen bonds between the truncated CAPN6 and VEGFA.

Additionally, a previous study suggests that CAPN6 plays an essential role in the process of ciliogenesis [16]. Deficiency of this protein leads to imbalances in this process [39]. Ciliogenesis regulates the formation of cilia, which are microtubule-based organelles present on the surface of various cell types, including those in the placenta. Notably, VEGFA has been identified as a key signaling pathway in physiological angiogenesis and is also a major therapeutic target [40]. Exploring the interaction between CAPN6 and VEGFA could be valuable for pharmacogenetic applications.

This study is primarily based on genomic and bioinformatic analyses, and therefore has inherent limitations. No functional assays were performed to directly assess the impact of the identified *CAPN6* variant on gene or protein expression, protein–protein interactions, or downstream signaling pathways. In particular, experimental approaches such as quantitative RT-PCR, Western blot analysis, or functional interaction studies (e.g., co-immunoprecipitation) were not conducted to evaluate the effects of the variant on CAPN6 expression or its interaction with VEGFA. Furthermore, the absence of placental tissue limited direct investigation of placental-specific mechanisms. Consequently, these findings should be considered exploratory and hypothesis-generating, and future functional studies in appropriate cellular and animal models will be essential to validate the proposed pathogenic mechanisms.

By addressing a current gap in the literature concerning CAPN6, this research endeavors to contribute significantly to our understanding of placental function and its impact on pregnancy outcomes. Functional validation studies will be essential to further clarify the role of *CAPN6* in neurodevelopmental disorders.

## 4. Materials and Methods

### 4.1. Library Preparation and NGS Analysis

Genomic DNA was retrieved from the patient’s and parents’ peripheral blood leukocytes, as previously described [41]. Library preparation (TRIOS) and exome enrichment were performed employing the Agilent SureSelect V7 kit (Santa Clara, CA, USA) following the manufacturer’s instructions. The sequencing run was performed using the Illumina HiSeq 3000 instrument (San Diego, CA, USA). This specific method allowed the achievement of 97% of regions covered at least 20×. The identified variants were filtered based on (i) recessive/de novo/X-linked pattern of inheritance, and (ii) allele frequencies (minor allele frequency, MAF) < 1% using as reference the following genomic datasets: 1000 Genomes, ESP6500, ExAC, and GnomAD, with HG38 as the reference genome, as commonly applied in clinical NGS diagnostics [42]. The confirmation of the de novo event was performed through conventional Sanger sequencing using the BigDyeTM Terminator v1.1 Cycle Sequencing Kit (Life Technologies, Carlsbad, CA, USA) with the SeqStudio Genetic Analyzer instrument (Thermo Fisher Scientific, Waltham, MA, USA). The sequences of the primers adopted for the Sanger confirmation were for. 5′-CTGCAGGAAGGTATCACGGT-3′, rev. 5′-ATTGGGGCTTCTACATGGAACT-3′. In alignment to a previous protocol, DNA fingerprint analysis was carried out to confirm maternity and paternity for both patient and parents [43].

### 4.2. Data Analysis

The variant was searched on the Human Gene Mutation Database (HGMD Professional 2025) and on the gnomAD database (https://gnomad.broadinstitute.org/) (accessed on 26 May 2025). Furthermore, it was filtered employing VarAft (2.17-2) [44]. The described variant was classified in alignment with the American College of Medical Genetics (ACMG) guidelines throughout VarSome, consistent with previous documentation [45]. The criteria adopted for variant classification were enumerated in Table 2.

Mutation taster (https://www.mutationtaster.org/) (accessed on 26 May 2025) was used to predict the pathogenic effect of the variant described. CAPN6 gene expression patterns of placenta tissues were obtained from the Human Protein Atlas (HPA) database (https://www.proteinatlas.org/) (accessed on 26 May 2025). Additionally, CAPN6 placental expression data were obtained from the GSE9984 microarray dataset [46], available in the Gene Expression Omnibus (GEO) database (https://www.ncbi.nlm.nih.gov/geo) (accessed on 26 May 2025). Data processing was performed in RStudio version 3.4.3 using the affy and oligo packages.

Gene ontology terms (GO) related to the protein and functional domain annotations were obtained from the QuickGO database (https://www.ebi.ac.uk/QuickGO/) (accessed on 26 May 2025), in addition to Uniprot (https://www.uniprot.org/) (accessed on 6 June 2024), InterPro (https://www.ebi.ac.uk/interpro/) (accessed on 26 May 2025) and Prosite (https://prosite.expasy.org/) (accessed on 26 May 2025) databases.

The online tool Mutalyzer 3.0 (https://mutalyzer.nl/) (accessed on 26 May 2025) was used to predict the amino acid sequence as a consequence of the mutation.

Protein structure predictions were generated using the AlphaFold3 server (https://alphafoldserver.com/, accessed on 26 May 2025), with five models produced for each protein. The model with the highest average predicted Local Distance Difference Test (pLDDT) score was selected for further analysis. All calculations were performed in RStudio using the bio3d, jsonlite, and ggplot2 packages. The Figure S7 presents line plots comparing the pLDDT scores of wild-type and mutant CAPN6 models. The average pLDDT confidence scores for the wild-type models (models 0–4) were 89.403, 89.333, 89.122, 89.143, and 89.152, respectively. For the mutant CAPN6 protein, the average pLDDT scores across the five models were 87.740, 87.978, 87.788, 87.829, and 87.684.

Additionally, AlphaFold3 was used to predict protein–protein interaction models between CAPN6 (both wild-type and mutant forms) and VEGFA. As before, the top-ranking model based on average pLDDT was selected for each condition. Figure S8 includes line plots visualizing pLDDT distributions for these interaction models. All the models selected were modeled and edited using UCSF ChimeraX (version 1.8, Resource for Biocomputing, Visualization, and Bioinformatics—RBVB, University of California, San Francisco), as described in a previous study [45,47].

## 5. Conclusions

In this study, we analyzed a family in which three affected siblings exhibit distinct neurodevelopmental and neuropsychiatric disorders, while the clinical phenotype of the mother remains to be fully characterized. Patient 1, the eldest male, presents with moderate intellectual disability accompanied by a schizophrenia spectrum disorder. Patient 2, the middle female sibling, exhibits an SLD affecting reading, writing, and arithmetic skills, along with recurrent headaches and anxiety-related internalizing symptoms. Patient 3, the youngest male, is affected by BIF and an emotional and behavioral disorder characterized by marked oppositional-defiant traits, language disorder (LD), and developmental coordination disorder (DCD), with significant hyperkinesia and hyperactivity. The mother has FIB and SLD with anxiety and depression disorder. WES identified a pathogenic variant in the CAPN6 gene, found in hemizygous condition in the affected brothers and in heterozygous condition in the mother and daughter. The variant was classified as pathogenic according to ACMG criteria and resulted in truncation of the CAPN6 protein within Domain III, a region critical for interaction with VEGF. In silico protein structure prediction revealed significant alterations in the mutated CAPN6 protein and its interaction with VEGF, caused by the identified mutation. These findings are exploratory and hypothesis-generating, underscoring the need for further investigation using advanced animal models and well-designed human studies to clarify gene–environment interactions during pregnancy and their potential contribution to the developmental origins of adult-onset mental disorders.

## Figures and Tables

**Table 1:** Summary of phenotypic features of the examined individuals.

Characteristic	Patient 1 (Male)	Patient 2 (Female)	Patient 3 (Male)	Mother
Gestational Age	37 weeks	38 weeks	37 weeks + 5 days	NA
Birth Weight/Length/HC	3050 g/49.5 cm/34 cm	2800 g/48 cm/33.5 cm	3150 g/47 cm/33.5 cm	NA
Developmental Milestones	GDD (walking at 18 mo, babbling at 12 mo, first words at 2 years)	GDD (walking at 16 mo, babbling at 12 m, first words at 2 years)	GDD (walking at 17 mo, minimal language development)	
Muscle Tone at First Evaluation	Reduced (generalized hypotonia, mild hypotrophy, kyphoscoliosis)	Normal	Normal	
EEG Findings	EEG at 7 years: central spikes during sleep	Normal	Normal	
Neurodevelopmental Trajectory	Initial: GDD	Initial: GDD	Initial: GDD	Initial: Normal acquisition of psychomotor developmental milestones.
	At 7 years: BIF and psychotic traits	At 5 years: SSD	At 7 years: BIF, ODD, LD, DCD	At 7 Years: Initial learning difficulties
	At 10 years: MoID, emergence of self-injurious behaviors, bizarre conduct, and psychotic traits. At 18 years: MoID with SSD	At 14 years: SLD affecting reading, writing, and arithmetic		At 11 years: BIF and LD
Behavioral Profile	Self-/other-directed aggression, poor adherence to social norms, oppositional traits, psychotic features	Anxiety features with internalizing symptoms	Irritability, oppositional and provocative behavior, hyperkinesia and hyperactivity	Anxiety and depression disorder
Somatic Traits	Marfanoid habitus, persistent microcephaly, distinctive craniofacial features, macroorchidism	Hypoplasia of the fourth metatarsal	Plagiocephaly	

Abbreviations. BIF: borderline intellectual functioning; DCD: developmental coordination disorder; EEG: electroencephalogram; GDD: global developmental delay; HC: head circumference; LD: language disorder; MoID: moderate intellectual disability; NA: not available; ODD: oppositional defiant disorder; SLD: specific learning disorders; SSD: schizophrenia spectrum disorder.

**Table 2:** ACMG guidelines adopted for the classification of the identified variant as pathogenic.

Criteria for Classifying Variants	Category Code	Description
Moderate	PM2	Absent from controls (or at extremely low frequency if recessive) in Exome Sequencing Project, 1000 Genomes Project, or Exome Aggregation Consortium.
Supporting	PP1	Cosegregation with disease in multiple affected family members in a gene definitively known to cause the disease.
Pathogenic strong	PVS1	Null variant (nonsense, frameshift, canonical ±1 or 2 splice sites, initiation codon, single or multiexon deletion) in a gene where LOF is a known mechanism of disease.
ACMG variant classification		Pathogenic

## Data Availability

The original contributions presented in this study are included in the article/Supplementary Materials. Further inquiries can be directed to the corresponding authors.

## Supplementary Materials

The following supporting information can be downloaded at: https://www.mdpi.com/article/10.3390/ijms27031140/s1.

## Abbreviations

The following abbreviations are used in this manuscript:

ACMG	American College of Medical Genetics
BIF	Borderline intellectual functioning
DCD	Developmental coordination disorder
EEG	Electroencephalogram
GDD	Global developmental delay
HC	Head Circumference
HGMD	Human Gene Mutation Database
LD	Language disorder
MAF	Minor allele frequency
MoID	Moderate intellectual disability
NMD	Nonsense mediated decay
ODD	Oppositional Defiant Disorder
SLD	Specific learning disorder
SSD	Speech Sound Disorder
VEGFA	Vascular endothelial growth factor
WES	Whole-exome sequencing
