# Supplementary material for: Potential Link Between a Disruptive CAPN6 Variant and Neurodevelopmental Disorders
Source: Int J Mol Sci. 2026 Jan 23;27(3):1140. doi: 10.3390/ijms27031140 (PMC12897768; doi:10.3390/ijms27031140)
Supplement: Supplementary file 1 [file ijms-27-01140-s001.zip › ijms-4087991-supplementary.pdf]

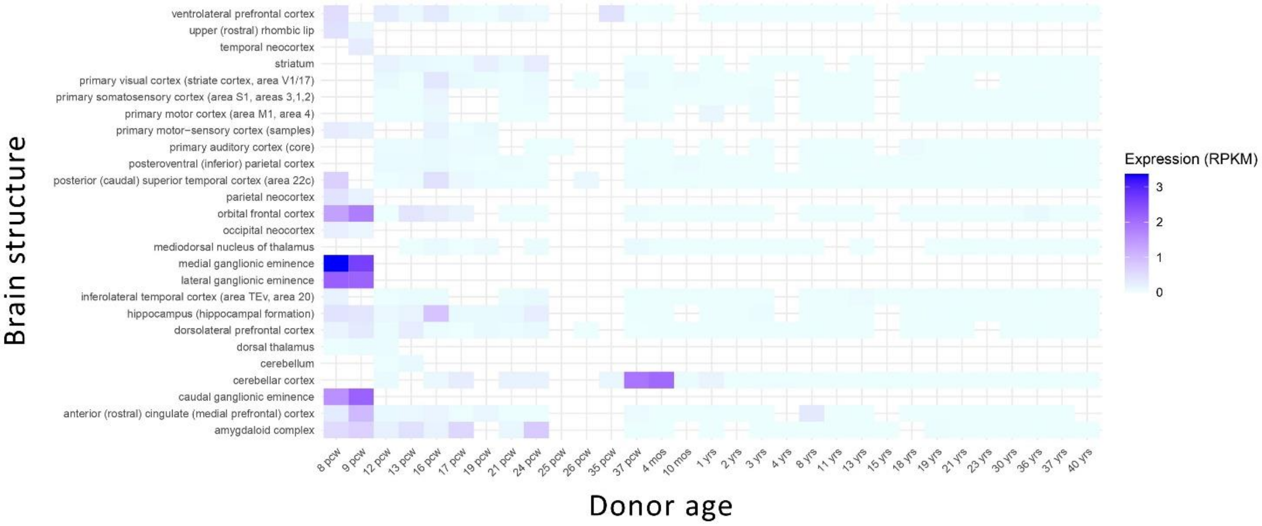

Figure S1. Heatmap showing *CAPN6* expression across various human brain regions. Expression levels are reported in reads per kilobase per million mapped reads (RPKM) and were obtained from the BrainSpan database. The heatmap was generated in RStudio (version 3.4.3) using the ggplot2 and pheatmap packages.

Average nTPM: 80.4

**Placenta sample 200**	
**Female; Age NA**	
nTPM	137.2
**Cell types**	**Percentage (%)**
Trophoblastic cells	55
Endothelial cells	25
Other cell types	20

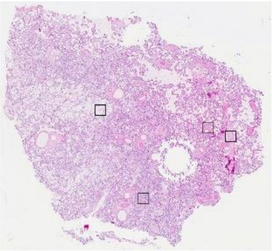

**Placenta sample 202**	
**Female; Age 28**	
nTPM	111
**Cell types**	**Percentage (%)**
Trophoblastic cells	45
Endothelial cells	5
Other cell types	50

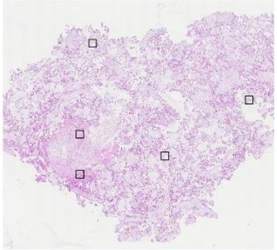

**Placenta sample 203**	
**Female; Age 39**	
nTPM	98.8
**Cell types**	**Percentage (%)**
Trophoblastic cells	45
Endothelial cells	35
Other cell types	20

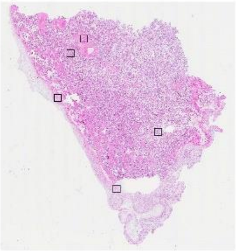

**Placenta sample 204**	
**Female; Age 31**	
nTPM	45.1
**Cell types**	**Percentage (%)**
Trophoblastic cells	40
Endothelial cells	35
Other cell types	25

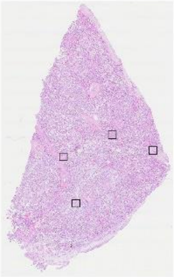

**Placenta sample 375**	
**Female; Age 30**	
nTPM	70.7
**Cell types**	**Percentage (%)**
Trophoblastic cells	55
Endothelial cells	20
Other cell types	25

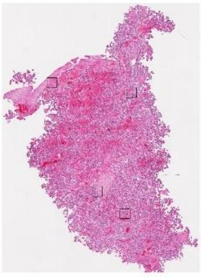

**Placenta sample 385**	
**Female; Age 31**	
nTPM	80.1
**Cell types**	**Percentage (%)**
Trophoblastic cells	50
Endothelial cells	20
Other cell types	30

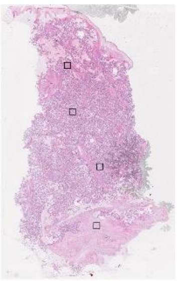

**Placenta sample 398**	
**Female; Age 37**	
nTPM	71.5
**Cell types**	**Percentage (%)**
Trophoblastic cells	50
Endothelial cells	20
Other cell types	30

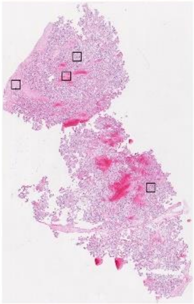

**Placenta sample 413**	
**Female; Age 36**	
nTPM	28.9
**Cell types**	**Percentage (%)**
Trophoblastic cells	45
Endothelial cells	10
Other cell types	45

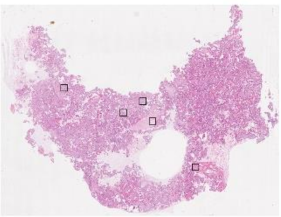

Figure S2. Placental expression of *CAPN6* across different tissue samples from the Human Protein Atlas (HPA) database. Expression levels are reported in normalized transcripts per million (nTPM). For each sample, donor age, sample ID, and the relative proportion of placental cell types are also indicated.

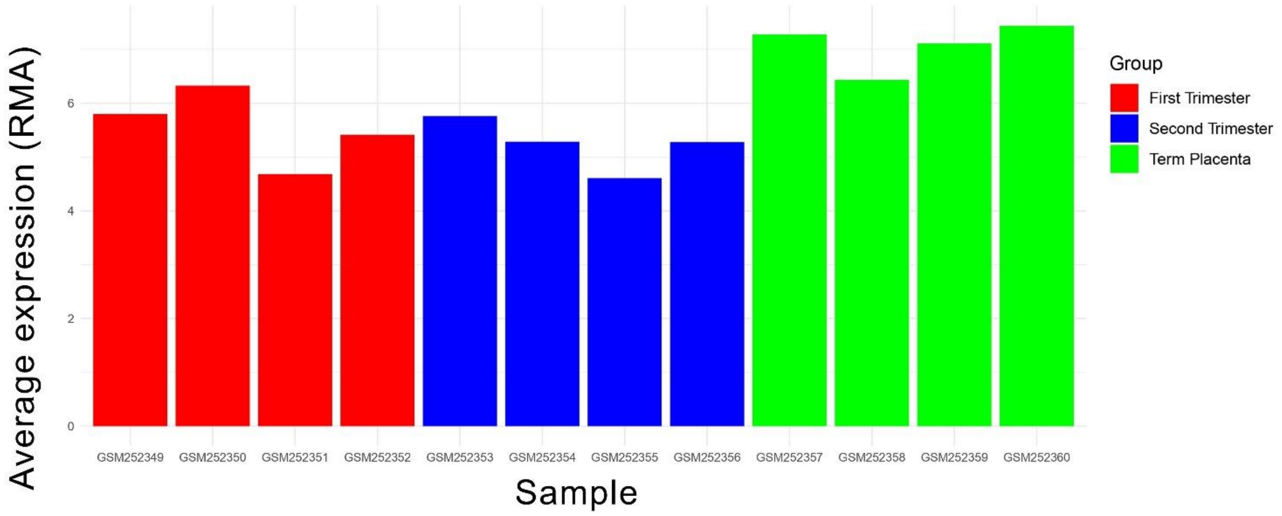

Figure S3. Placental *CAPN6* gene expression across three gestational stages: first trimester, second trimester, and term. Expression profiling was performed using Affymetrix microarray data retrieved from the GSE9984 dataset (Gene Expression Omnibus). Data were processed and visualized using the affy, oligo, and ggplot2 packages in RStudio (version 3.4.3).

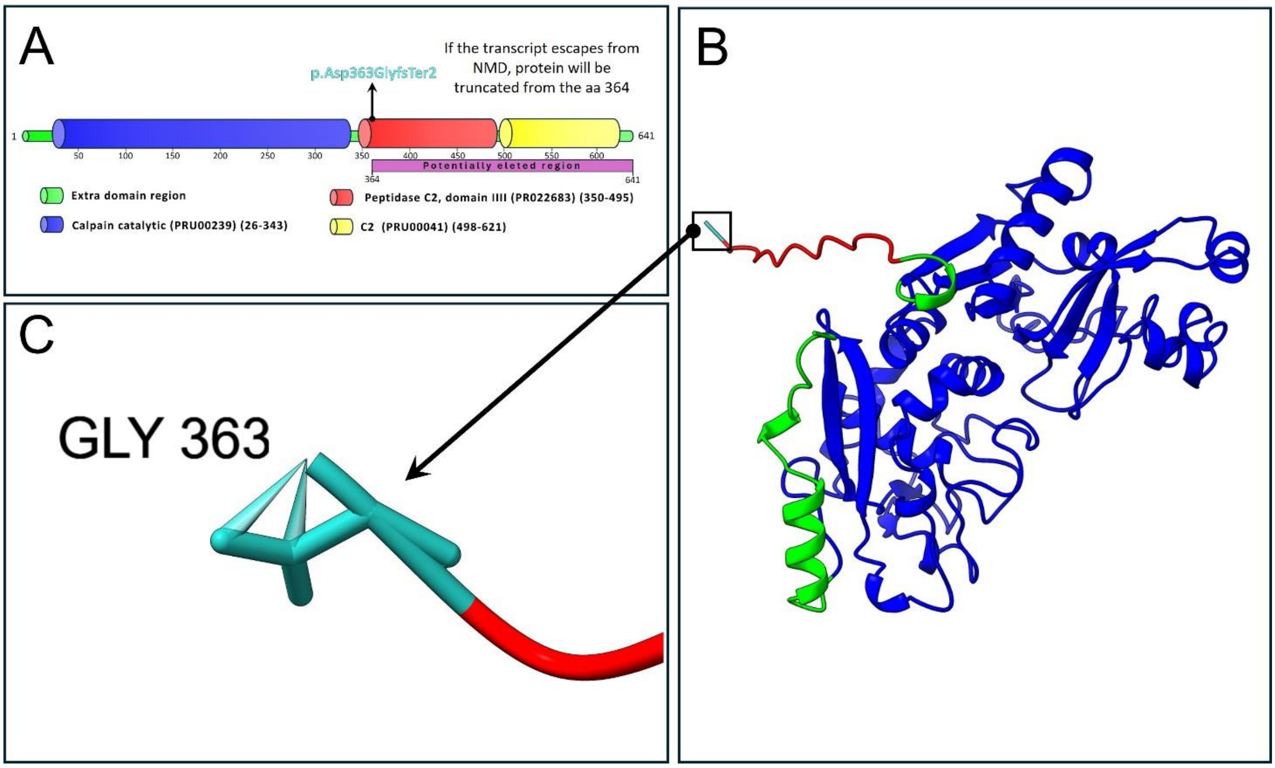

Figure S4. Protein structure prediction of the truncated CAPN6 protein carrying the p.Asp363GlyTer2 mutation. (A) Schematic representation of CAPN6 domain organization with functional annotations from InterPro and ProSite. (B) Predicted 3D structure of the truncated CAPN6 protein (p.Asp363GlyTer2), highlighting the truncation site (black square). Domain colors correspond to those in panel (A). (C) Close-up view of the mutated residue Gly363, beyond which the protein is truncated. Structural predictions were generated using the AlphaFold3 server and visualized with UCSF ChimeraX.

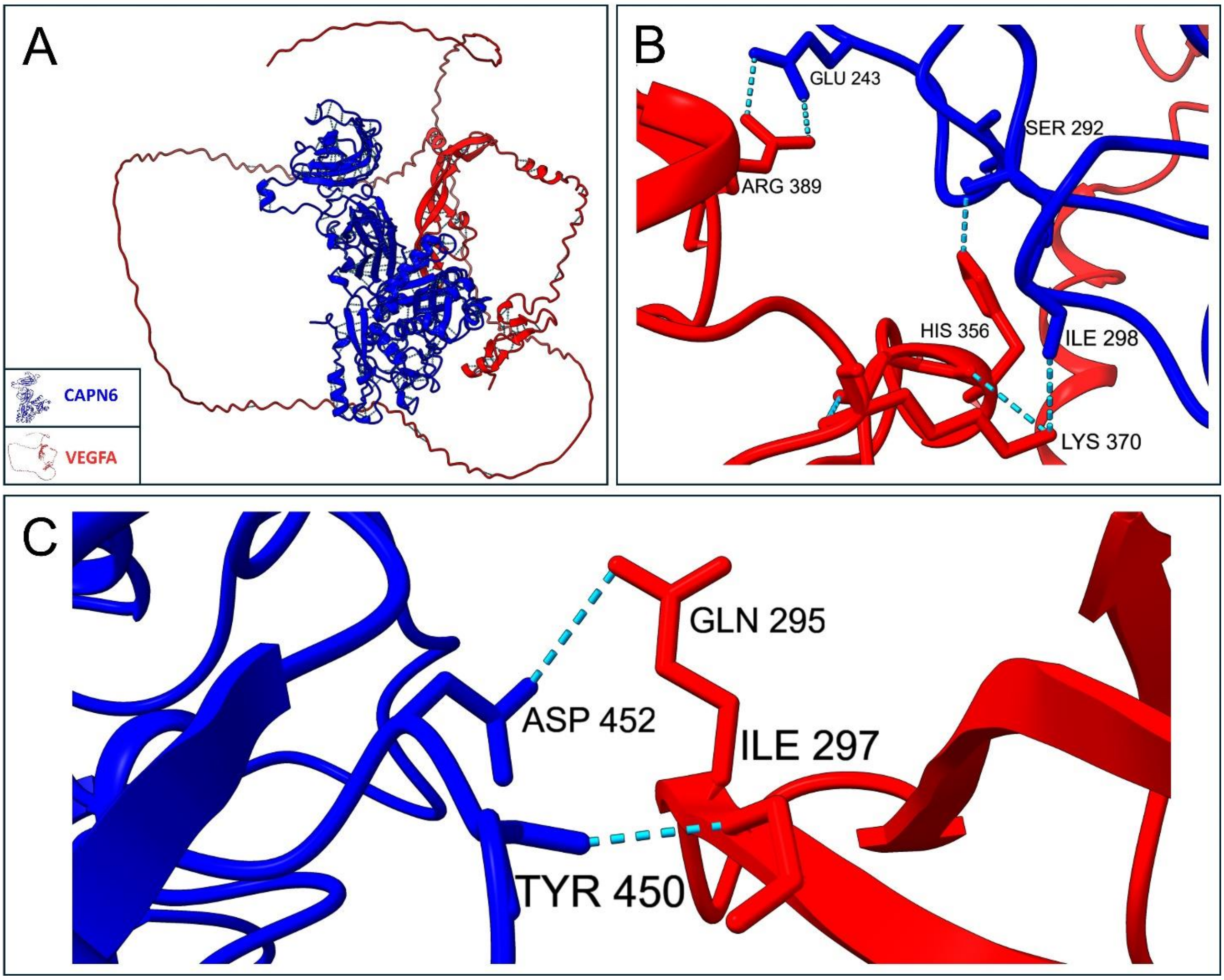

Figure S5. Predicted molecular interaction between wild-type CAPN6 and VEGFA. (A) Structural overview of the predicted CAPN6 (blue)-VEGFA (red) complex, demonstrating potential binding interfaces between the proteins. (B) Detailed view of the interaction site showing hydrogen bonds between CAPN6 residues (Glu243, Ser292, Ile298) and VEGFA residues (Arg389 [double bond], His366, Lys370). (C) Additional interaction interface featuring hydrogen bonds between CAPN6 (Tyr450, Asp452) and VEGFA (Ile297, Gln295). All structural predictions were visualized and analyzed using the molecular modeling software UCSF ChimeraX version 1.8.

Table S1. Hydrogen bonds of the best model predicted for the interaction between the wild type CAPN6 and VEGFA. Atomic distance has been expressed in Angstrom (Å).

Protein 1	Donor residue	Donor atom	Protein 2	Acceptor residue	Acceptor atom	Distance (Å)
CAPN6	Ser292	OG	VEGFA	His356	NE2	2.638
VEGFA	Gln295	NE2	CAPN6	Asp452	OD2	3.365
VEGFA	Ile297	N	CAPN6	Tyr450	O	2.769
VEGFA	His356	NE2	CAPN6	Ser292	OG	2.638
VEGFA	Lys370	NZ	CAPN6	Ile298	O	3.308
VEGFA	Arg389	NH1	CAPN6	Glu243	OE1	2.351
VEGFA	Arg389	NH2	CAPN6	Glu243	OE2	1.802

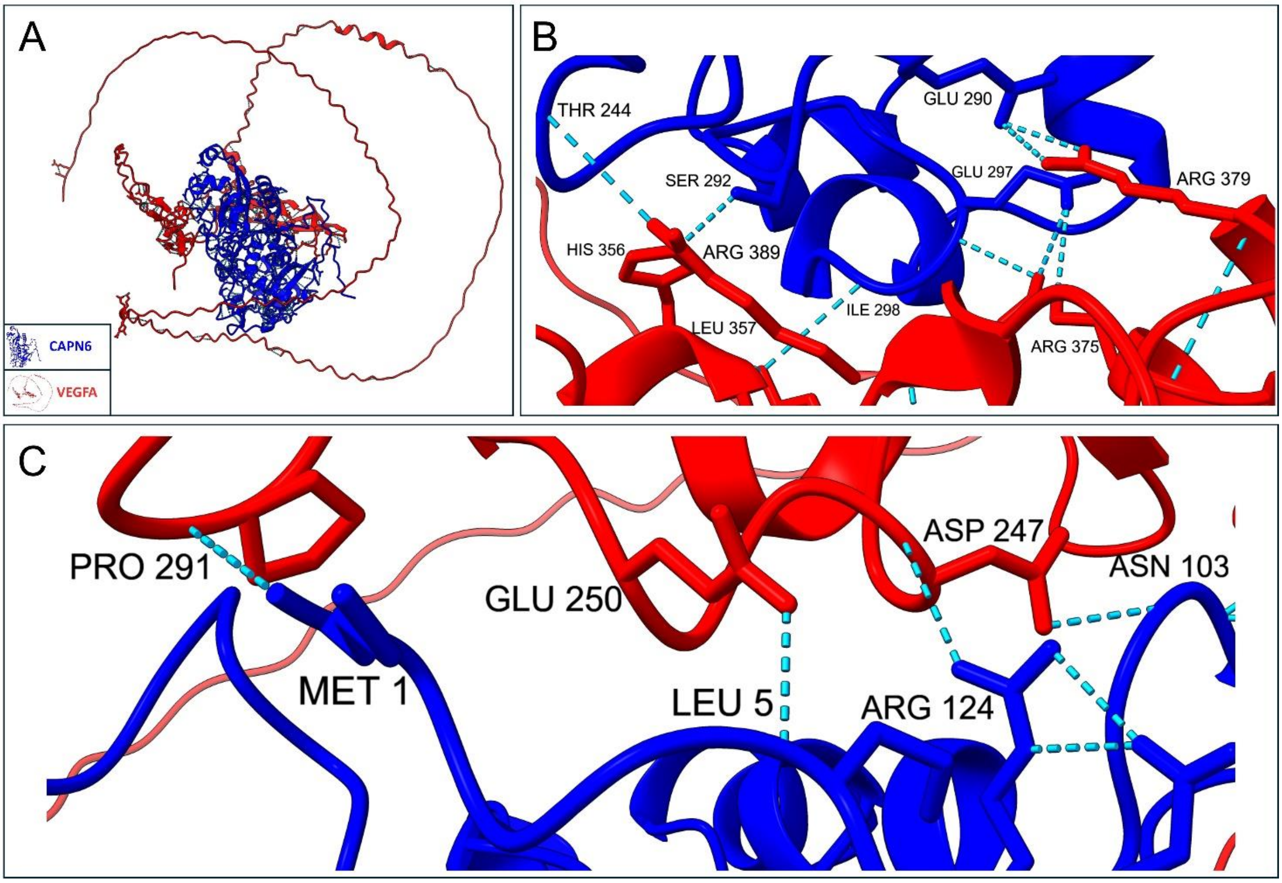

Figure S6. Predicted interaction of truncated CAPN6 (p.Asp363GlyfsTer2) with VEGFA. (A) Structural overview showing altered binding interfaces compared to wild-type. (B) Close-up of mutant-specific hydrogen bonds between the CAPN6 residues Thr244, Glu290, Ser292, Glu297 and Ile298, and VEGFA Arg389, Arg379 (2 bonds), His356, Arg375 (3 bonds) and Leu357, respectively. (C) Hydrogen bonds interaction site involving CAPN6 (Met1, Leu5, Asn103, Arg124) and VEGFA (Pro291, Glu250, Asp247). All structures were computationally modelled using UCSF ChimeraX version 1.8 starting from the AlphaFold3 best model selected.

Table S2. Hydrogen bonds of the best model predicted for the interaction between the mutated p.Asp363GlyfsTer2 CAPN6 and VEGFA. Atomic distance has been expressed in Angstrom (Å).

Protein 1	Donor residue	Donor atom	Protein 2	Acceptor residue	Acceptor atom	Distance (Å)
CAPN6	Met1	N	VEGFA	Pro291	O	2.082
CAPN6	Leu5	N	VEGFA	Glu250	OE1	3.039
CAPN6	Asn103	N	VEGFA	Asp247	OD1	3.097
CAPN6	Arg124	NH1	VEGFA	Asp247	O	2.762
CAPN6	Ser292	OG	VEGFA	His356	NE2	2.887
VEGFA	His356	NE2	CAPN6	Ser292	OG	2.887
VEGFA	Lys370	NZ	CAPN6	Ile298	O	3.441
VEGFA	Arg375	NE	CAPN6	Glu297	OE2	3.137
VEGFA	Arg375	NH2	CAPN6	Glu297	O	3.359
VEGFA	Arg375	NH2	CAPN6	Glu297	OE2	2.92
VEGFA	Arg379	NH1	CAPN6	Glu290	OE2	2.651
VEGFA	Arg379	NH2	CAPN6	Glu290	OE2	2.825
VEGFA	Arg389	NH2	CAPN6	Thr244	O	2.773

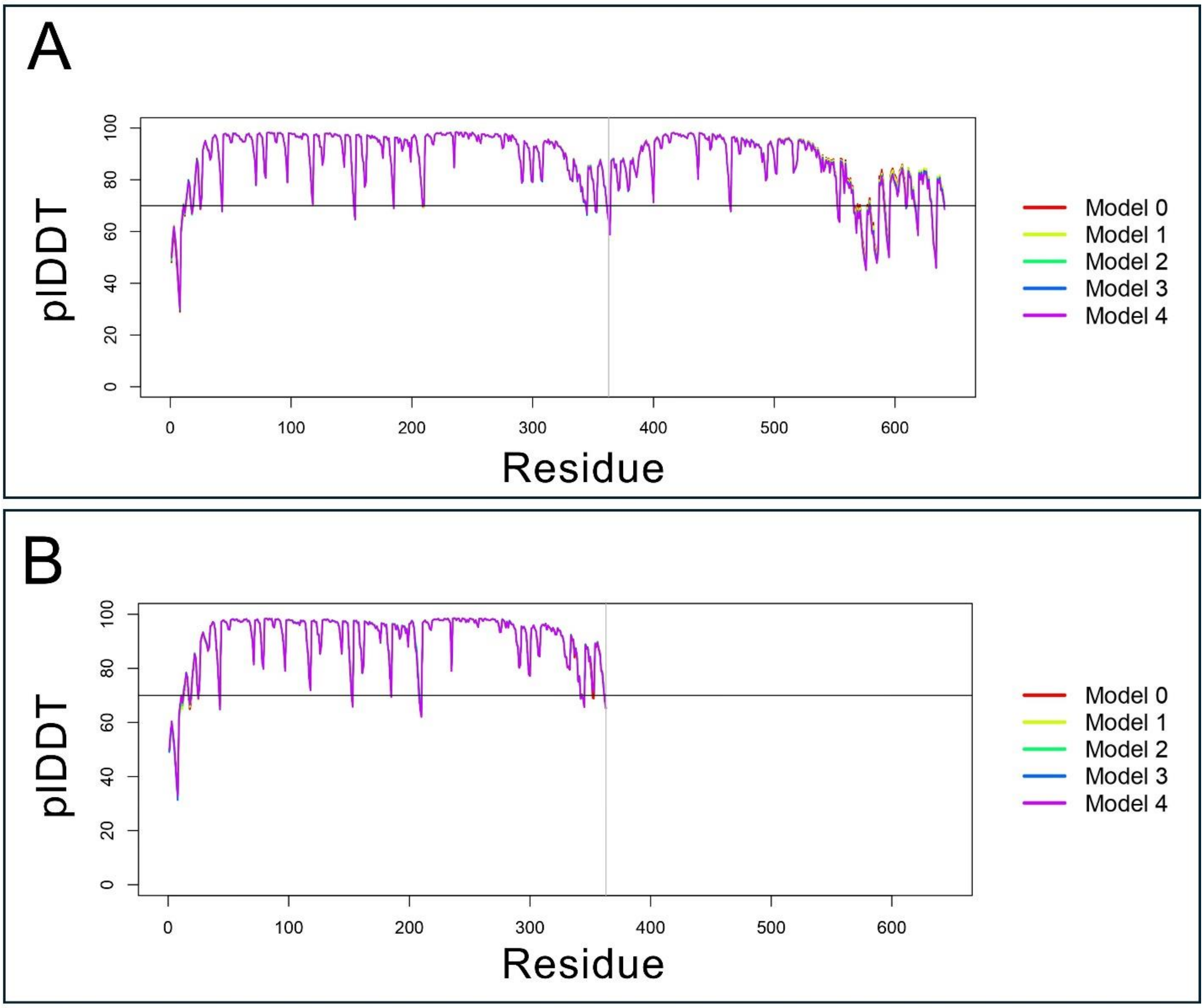

Figure S7. Line plot showing the plDDT variation across the wild-type and mutated CAPN6 AlphaFold3 prediction models. (A) plDDT values across all residues of the wild-type CAPN6 protein, with the top-ranked model (model 0) displaying an average plDDT of 89.4. (B) plDDT variation in the mutated CAPN6 protein (p.Asp363GlyTer2), with the top-ranked model (model 1) showing an average plDDT of 88.0 across the predicted residues.

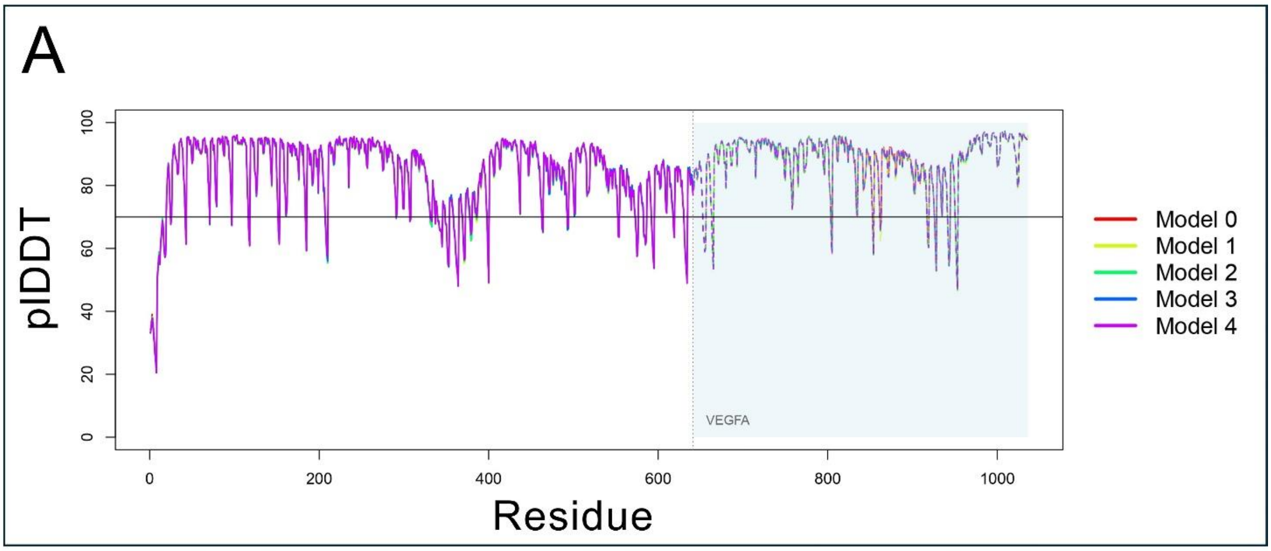

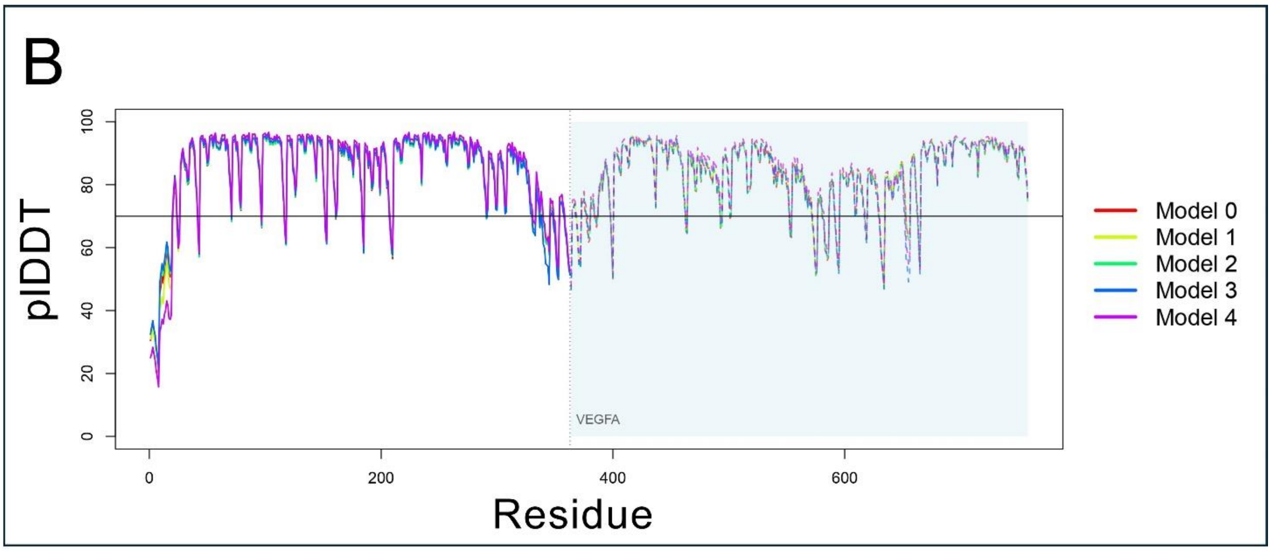

Figure S8. Line plot showing plDDT variation across the AlphaFold3-predicted protein–protein interaction models between CAPN6 (wild-type and mutant) and VEGFA. (A) plDDT variation across the AlphaFold3 models of the interaction between wild-type CAPN6 and VEGFA. The top-ranked model was Model 0, with an average plDDT score of 89.403. (B) plDDT variation across the AlphaFold3 models of the interaction between mutant CAPN6 (p.Asp363GlyTer2) and VEGFA. The top-ranked model was Model 1, with an average plDDT score of 87.978.
